# Serum Ghrelin and Leptin Concentrations in Patients with Major Depressive Disorder before and after Supplementation with Vitamin D3

**DOI:** 10.1155/2024/2057881

**Published:** 2024-03-25

**Authors:** Shareefa AlGhamdi, Nabilah Alsulami, Sawsan Khoja, Hadeil Alsufiani, Haythum O. Tayeb, Huda Alshaibi, Frank I. Tarazi

**Affiliations:** ^1^Department of Biochemistry, Faculty of Sciences, King Abdulaziz University, Jeddah, Saudi Arabia; ^2^Vitamin D Pharmacogenomics Research Group, King Abdulaziz University, Jeddah, Saudi Arabia; ^3^Experimental Biochemistry Unit, King Fahd Medical Research Center, King Abdulaziz University, Jeddah, Saudi Arabia; ^4^The Mind and Brain Studies Initiative, The Neuroscience Research Unit, Division of Neurology, Department of Internal Medicine, Faculty of Medicine, King Abdulaziz University, Jeddah, Saudi Arabia; ^5^Embryonic Stem Cell Unit, King Fahd Medical Research Center, King Abdulaziz University, Jeddah, Saudi Arabia; ^6^Department of Psychiatry and Neuroscience Program, Harvard Medical School and McLean Hospital, Boston, MA, USA

## Abstract

**Aim:**

To determine serum concentrations of leptin and ghrelin in patients with major depressive disorder (MDD) before and after vitamin D3 supplementation.

**Methods:**

A total of 72 participants were recruited in this study (40 MDD patients and 32 healthy controls). MDD was diagnosed by using Beck's Depression Inventory (BDI) scale. Blood samples were collected from all participants at the beginning of the study to determine baseline serum 25(OH)D3, leptin, and ghrelin concentrations. Patients were then treated weekly with vitamin D3 (50,000 IU) for 3 months, and blood samples were collected again by the end of the study.

**Results:**

At baseline, serum leptin concentrations were significantly higher in MDD patients than in healthy controls. In contrast, serum ghrelin concentrations were significantly lower compared to those in healthy controls. After supplementation with vitamin D3 for three months, MDD patients showed improvements characterized by a decrease in their BDI's scores and an increase in their serum vitamin D and ghrelin concentrations. No effects of vitamin D3 supplementation were seen on serum leptin concentration.

**Conclusions:**

The antidepressant effects of vitamin D3 supplementation could be mediated by ghrelin but not leptin.

## 1. Introduction

Major depressive disorder (MDD) is one of the most prevalent mental illnesses that negatively affects how a person feels, thinks, and acts. MDD can significantly impact patients' ability to function in daily life, affecting their relationships, work, and overall well-being. The severity of MDD symptoms can vary ranging from mild to severe and may include difficulty concentrating, feelings of worthlessness or guilt, changes in appetite, sleep disturbances, and fatigue. MDD is a multifactorial disorder that can be caused by a variety of psychological, social, and biological factors [1]. The biological factors include genetics, inflammatory biomarkers, hypothalamic-pituitary-adrenal (HPA) axis dysfunction, and physical health conditions as well as neurotransmitters and hormonal imbalances [1].

Leptin is a hormone encoded by the obese gene and released by adipose tissues [2]. While leptin's function is well established in appetite regulation and energy homeostasis, it seems to play a significant role in the regulation of the central nervous system (CNS) and contributes to the pathophysiology of several neurological diseases including mood disorders [3–5]. Leptin receptors are found to be expressed in many brain regions, such as the cortex and the nucleus of the solitary tract, hippocampus, the arcuate nucleus of the hypothalamus, olfactory bulb, and the dorsal raphe nucleus [6]. Deletion of leptin receptor was reported to induce depressive-like behaviors, suggesting that leptin signaling may be involved in the molecular mechanisms mediating the antidepressant effects of leptin [7]. It has been reported that injection of leptin into the hippocampus demonstrated an antidepressant-like effect in the forced swim test indicated by increasing swimming time and decreasing immobility, while injection of leptin into the hypothalamus showed no effect [8]. Human studies investigating the relationship between leptin and depression yielded inconsistent results. Lower leptin concentrations were reported by Kraus et al. in MDD patients compared with healthy controls [9], while Milaneschi et al. reported higher leptin concentration in MDD patients than controls [10]. Other studies found no differences in serum leptin concentrations between MDD patients and healthy controls [11, 12].

Ghrelin is a peptide hormone released by the endocrine cells of the stomach and the neurons in the arcuate nucleus of the hypothalamus [13]. Although ghrelin's key role in regulating appetite is well established, its involvement in stress, anxiety, and depression remains under active investigation. Ghrelin receptors are expressed in the hippocampus, amygdala, ventral tegmental area (VTA), and nucleus accumbens [14]. Animal studies reported a dual role for ghrelin; few studies revealed that ghrelin could be an anxiogenic-like hormone whereas others suggested that it can have anxiolytic-like and antidepressive-like responses [15–17]. The relationship between depression and ghrelin was also investigated in humans, and the results were inconsistent. Higher serum ghrelin concentrations were reported in MDD patients compared to healthy controls [11, 18, 19]. In contrast, other studies reported no differences or lower serum ghrelin concentration in patients with depression compared to controls [20, 21].

Several human studies revealed that serum leptin and ghrelin concentrations were affected by antidepressant drugs. For example, mirtazapine was reported to increase serum leptin levels and decrease serum ghrelin levels [9, 22, 23]. Furthermore, amitriptyline and maprotiline were found to increase serum leptin and ghrelin concentrations, respectively [23, 24]. Several studies have found a lower serum vitamin D in patients with depression compared to controls [25, 26]. Recently, few human studies have investigated vitamin D3 supplementation as a potential treatment for depression. Results showed that vitamin D3 supplementation ameliorated severity of MDD symptoms [27, 28]. On the other hand, published studies about vitamin D effect on leptin synthesis and secretion are contradictory. Kong et al. have demonstrated an increased expression of leptin mRNA and protein synthesis in adipose tissue culture through VDR suggesting both direct and indirect effect of vitamin D on leptin [29]. On contrast, Menendez et al. showed that treatment of human adipose tissue cultures with vitamin D inhibited leptin secretion [30]. However, it is still not clear whether the antidepressive-like effects of vitamin D3 supplementation on MDD are mediated by ghrelin and leptin or not. We hypothesized that ghrelin and leptin participate in the antidepressive-like effect of vitamin D3. There are very few studies on the association of vitamin D with leptin and ghrelin particularly in individuals with MDD. Therefore, the aim of the present study is to determine the serum concentrations of leptin and ghrelin in MDD patients before and after vitamin D3 supplementation.

## 2. Materials and Methods

This study was designed as an interventional study and approved by the Unit of Biomedical Ethics at the Faculty of Medicine at King Abdulaziz University, Jeddah, Saudi Arabia (Ref no. 30-18/Feb. 2018). A total of 72 adult participants were recruited. G∗Power 3.1 software was used to calculate the required sample size given power (95%), *α* (5%), and effect size (0.83). Forty patients diagnosed with MDD were recruited from the psychiatry clinic at King Abdulaziz University Hospital. Diagnosis of MDD was established at the psychiatry clinic using DSM-5 criteria, and the severity of MDD was evaluated using the Beck's Depression Inventory (BDI) test, the most widely used screening tool for depressive disorders that is also used to measure the severity of depression (Table 1). All patients received standard of care (SOC), which included treatment with selective serotonin reuptake inhibitors (SSRIs) and psychotherapy during the trial. Two questionnaires were used through a personal interview to check factors that can affect vitamin D levels like diet, sun exposure, a brief lifestyle, and demographic factors including age, gender, marital status, level of education, past medical history, and smoking status. This data was collected by a self-report questionnaire at the time of the clinic visit. The body mass index (BMI) was calculated at baseline and after a period of 3 months. The blood sample was withdrawn from each subject at the beginning of the study and at the end. The second questionnaire used was Beck's Depression Inventory (BDI) for evaluation of the severity of MDD. This group of patients was considered as intervention or treated group and was given vitamin D3 supplements (50,000 IU) for 3 months (one tablet/week). Thirty-two physically and mentally healthy participants (approved by DSM-5 test) were also recruited from King Abdulaziz University Hospital and considered as the control group. This group was recruited for baselines comparison, and they were not supplemented with vitamin D3. The exclusion criteria to select control group include abnormal serum PTH, age less than 18 and more than 65, patients with kidney and/or liver diseases, confirmed MDD according to DSM-5 criteria, and inability to provide consent. To ensure the control group fits the inclusion criteria, the health status of the control group was evaluated by measuring lipid profile, PTH, Ca, Mg, P, and TSH. All participants signed a form indicating their informed consent.

### 2.1. Anthropometric Measurements

Measurements were made in the morning with the participants wearing light clothing without shoes. Essential physical data including weight and height were measured using a digital scale. The BMI was then calculated by dividing the weight (kilogram) by the squared height (meter).

### 2.2. Biochemical Measurements

Blood samples (6 ml) were collected in the morning (between 9 and 11 am) after an overnight fasting on day one (baseline) and at the end of the study (after 3 months). The serum was separated and stored at -80°C until analysis.

Serum vitamin D was measured using a full automated system (Dimension Vista® System) at King Abdulaziz University Hospital. This assay is intended for the quantitative determination of 25 hydroxyvitamin D3 (25(OH)D3) in human serum and plasma. The measurement involves the competition principle: 1st incubation: by incubating the sample (15 *μ*l) with pretreatment reagent 1 and 2, bound vitamin D (25-OH) is released from vitamin D protein. 2nd incubation: by incubating the pretreated sample with the ruthenium-labeled vitamin D-binding protein, a complex between the vitamin D (25-OH) and the ruthenylated vitamin D-binding protein is formed. 3rd incubation: after addition of streptavidin-coated microparticles and vitamin D (25-OH) labeled with biotin, unbound ruthenium-labeled vitamin D-binding proteins 37 become occupied. A complex consisting of the ruthenylated vitamin D-binding protein and the biotinylated vitamin D (25-OH) is formed and becomes bound to the solid phase via interaction of biotin and streptavidin. The reaction mixture is aspirated into the measuring cell where the microparticles are magnetically captured onto the surface of the electrode. Unbound substances are then removed with Pro Cell/Pro Cell M. Application of voltage to the electrode then induces chemiluminescent emission which is measured by a photomultiplier. Results are determined via a calibration curve which is instrument specifically generated by 2-point calibration and a master curve provided via the reagent barcode.

Serum ghrelin was measured using ELISA kit (http://www.elabscience.com, Cat. No. E-EL-H1919: intra-assay CV% = 4.6%, interassay CV% = 5.8%) while serum leptin was measured using leptin ELISA kit by DBC (Diagnostics Biochem Canada) (Cat. No. CAN-L-4260: intra-assay CV% = 4.6%, interassay CV% = 5.8%). All kits used in this study were of analytical grade. In summary, ELISA kit uses competitive ELISA as the method. The microtiter plate provided in this kit has been precoated with an antigen specific to either human leptin or ghrelin. During the reaction, human leptin/ghrelin in the sample or standard competes with a fixed amount of human leptin/ghrelin on the solid phase supporter for sites on the biotinylated detection Ab specific to human leptin/ghrelin. Excess conjugate and unbound sample or standard were washed from the plate, and avidin conjugated to horseradish peroxidase (HRP) was added to each microplate well and incubated. Then, a TMB substrate solution was added to each well. The enzyme substrate reaction is terminated by addition of stop solution, and the color change was measured spectrophotometrically at wavelength of 450 nm. The concentration of human leptin/ghrelin in the samples was then determined by comparing the OD of the samples to the standard curve.

### 2.3. Statistical Analysis

Data analysis was carried out using the Statistical Package for the Social Sciences (SPSS) software (version 24, SPSS Inc., Chicago, IL, USA), while the software package GraphPad (version 6.0, Prism, CA, US) was used to plot graphs. Data were presented as mean ± standard error of mean (SEM). Independent samples *t*-test was used to compare the means for two independent groups such as the control group and patient group (either before or after vitamin D3 supplementation). On the other hand, paired sample *t*-test was used to determine the significant difference in mean of variables in patients before and after supplementation with vitamin D3. A *p* value of 0.05 or lower was considered statistically significant.

## 3. Results

Forty MDD patients (8 males and 32 females) and forty one healthy controls (20 males and 21 females) participated in this study with a mean age of 42 and 28 years, respectively (Table 2). At baseline, MDD patients were obese with a mean BMI of 31 kg/m^2^ compared to controls with a mean BMI of 26 kg/m^2^. After supplementation with vitamin D3, BMI of MDD patients was nonsignificantly decreased.

**Figure 1:**
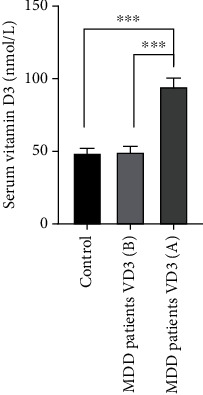
Mean serum 25(OH)D3 concentrations of healthy controls and patients with major depressive disorder MDD (at baseline (B) and after (A) supplementation with vitamin D3 for three months). Error bars show SEM. ⁣^∗∗∗^*p* < 0.001.

**Figure 2:**
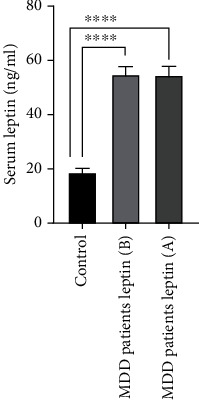
Mean serum leptin concentrations of healthy controls and patients with major depressive disorder MDD (at baseline (B) and after (A) supplementation with vitamin D3 for three months). Error bars show SEM. ⁣^∗∗∗∗^*p* < 0.0001.

**Figure 3:**
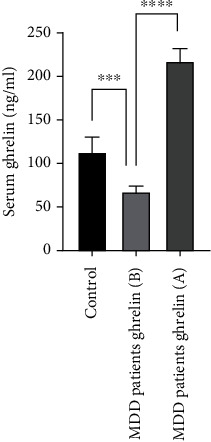
Mean serum ghrelin concentrations of healthy controls and patients with major depressive disorder MDD (at baseline (B) and after (A) supplementation with vitamin D3 for three months). Error bars show SEM. ⁣^∗∗∗^*p* < 0.001; ⁣^∗∗∗∗^*p* < 0.000.

At baseline, no significant difference between mean serum 25(OH)D3 concentration of MDD patients (50 ± 5.1 nM) and controls (49 ± 4.4 nM) was found (Figure 1). In contrast, mean serum leptin concentrations in MDD patients were higher (55 ± 3.5 ng/ml) than those in controls (19 ± 2.2 ng/ml) (Figure 2). Contrary to leptin, mean serum ghrelin concentrations in MDD patients were lower (67 ± 8.4 ng/ml) than those in controls (113 ± 19.3 ng/ml) (Figure 3).

**Figure 4:**
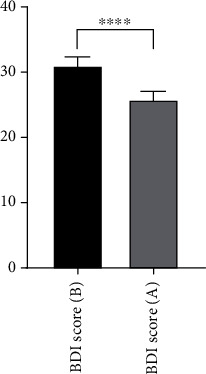
Mean Beck's Depression Inventory (BDI) scale of patients with major depressive disorder MDD (at baseline (B) and after (A) supplementation with vitamin D3 for three months). Error bars show SEM. ⁣^∗∗∗∗^*p* < 0.0001.

After vitamin D3 supplementation for three months, mean serum 25(OH)D3 concentration of MDD patients was significantly increased from 50 ± 4.4 to 95 ± 6.9 nM (Figure 1). Unlike serum 25(OH)D3, mean serum leptin concentration was not changed (Figure 2). In contrast to leptin, mean serum ghrelin concentrations were significantly increased from 67 ± 8.4 ng/ml to 217 ± 16.7 ng/ml after supplementation with vitamin D3 (Figure 3). These changes were found to be accompanied by a significant decrease in BDI scores in MDD patients from 31 ± 1.6 to 26 ± 1.5 ng/ml (Figure 4).

## 4. Discussion

The purpose of this study was to determine the level of serum leptin and ghrelin in MDD patients before and after vitamin D3 supplementation. At baseline, MDD patients had higher leptin concentrations and elevated BMI vs. control subjects. These results are consistent with the findings of Milaneschi et al. who reported that higher leptin levels were associated with the atypical MDD subtype cases [10]. This association was mediated by adiposity levels strengthening the hypothesis of the involvement of leptin resistance [31]. Supporting these results are the findings of a recent study showing that MDD patients are characterized by higher leptin levels [32]. In contrast, earlier studies reported lower leptin concentrations in MDD patients compared to healthy controls [9, 33]. In animal studies, leptin displays antidepressant effects [34, 35]. Other studies reported no differences in leptin levels between healthy controls and MDD patients [12]. This inconsistency may result from potentially confounding factors such as gender ratios, age, depression subtype, medication history, fat content, and other metabolic factors. Thus, in order to interpret leptin role in MDD more accurately, further studies are required to take into consideration these potentially confounding factors.

In contrast to leptin, MDD patients had lower serum ghrelin concentrations than healthy controls at baseline. The published clinical data regarding the levels of ghrelin in individuals with depression remains controversial. While certain studies have indicated that there are no alterations in the levels of ghrelin among depressed patients, others have reported either decreased or increased levels [19, 36]. Similar to our results, Barim et al. reported that MDD patients had lower serum ghrelin than healthy controls suggesting that ghrelin may be involved in the pathophysiology of MDD [20]. Interestingly, long-term stress has been found to elevate the levels of circulating acyl-ghrelin, and this increase persists even after the stressor has ceased [37]. It is hypothesized that the secretion of ghrelin may act as a counter-regulatory response to stress, and higher levels of ghrelin may be necessary to prevent excessive anxiety levels [38]. In contrast, another study examined ghrelin levels in patients with major depressive disorder (MDD) compared to healthy controls. The researchers found that individuals with MDD had significantly higher ghrelin levels than the control group, suggesting a dysregulation of ghrelin in depression [39]. Also, Lutter et al. suggest that the presence of ghrelin resistance, typically associated with obesity, can diminish the antidepressant and neuroprotective effects of ghrelin, thereby leading to symptoms of major depressive disorder (MDD) [37]. The inconsistency in studies reporting ghrelin levels in depressed patients may also be due to several factors including differences in patient's characteristics (such as age, gender, BMI, smoking, and depressive symptom severity), screening tools used, and the variability in ghrelin measurements methods. Collectively, based on the existing findings, it appears that ghrelin has an impact on mood. However, the intricate relationship between the nervous system, regulation of ghrelin, and mental disorders necessitates additional investigation to better understand this interplay. It is possible that the diverse clinical characteristics observed in major depressive disorder may play a role, at least partially, in the lack of conclusive findings.

After supplementation with vitamin D3 (50,000 IU) for three months, MDD patients showed improvements in their symptoms evidenced by a decrease in their BDI's score that correlated with an increase in serum vitamin D and ghrelin concentrations [27]. In contrast, vitamin D3 supplementation did not alter the levels of serum leptin. Vitamin D supplementation and its potential effects on depression have been the subject of several studies. Cross-sectional study and randomized double blind controlled trial conducted by Jorde et al. investigated the effect of vitamin D supplementation on symptoms of depression in overweight and obese subjects. The study found that participants who received vitamin D supplementation experienced a significant reduction in depressive symptoms compared to those who received a placebo [40].

To our knowledge, this is the first study that investigated the effect of vitamin D3 supplementation on ghrelin levels in MDD patients. The results showed that serum ghrelin concentration was significantly increased after vitamin D supplementation. Similarly, one study determined the effects of vitamin D-fortified doogh (a yogurt drink) on ghrelin levels in diabetic patients, and the results showed that daily intake of vitamin D-fortified doogh increased circulating ghrelin levels [41]. Our results are in agreement with animal studies suggesting that ghrelin displays an antidepressant-like effect. It is noteworthy that ghrelin receptors are present in the brain including regions such as the hippocampus, amygdala, ventral tegmental area (VTA), and nucleus accumbens [14], indicating a role for ghrelin mediating depressive-related behaviors [42]. Injection of ghrelin in mice was found to alleviate depressive-like behaviors stimulated by prolonged moderate stress [35]. It modulated depressive-related signals by forming neuronal networks with various neurotransmitter systems and neuropeptides [17]. Ghrelin was found to increase the release of dopamine in the nucleus accumbens and ventral tegmental areas [42]. When ghrelin binds to substantia nigra pars compacta (SNpc) cells, it activates SNpc dopamine neurons, leading to an increase in tyrosine hydroxylase mRNA and dopamine concentrations in the dorsal striatum [42].

In the current study, vitamin D supplementation resulted in no significant changes in leptin levels in MDD subjects. One possible explanation for the lack of an effect on leptin levels could be the use of antidepressant medications by the patients in our study. Previous studies have indicated that antidepressants can increase leptin levels [23, 43]. Therefore, the concurrent use of antidepressants may have masked the potential impact of vitamin D on leptin levels in our study population.

Overall, our findings contribute to the growing body of evidence supporting the beneficial effects of vitamin D in reducing depression. However, further investigation is warranted to better understand the complex interplay between vitamin D, depression, and leptin levels in different populations and under varying conditions.

## 5. Limitations

A significant limitation of this study is that patients were taking medications during this study for ethical reasons. These antidepressants might affect and/or interfere with the levels of measured biomarkers. This study is also limited by the intervention period (3 months).

## 6. Conclusions

The study found that serum leptin concentrations were higher in MDD patients, while serum ghrelin concentrations were lower compared to healthy controls. Furthermore, MDD patients showed improvements in their depressive symptoms characterized by decreasing their BDI's scores and increasing their serum vitamin D3 and ghrelin concentrations after supplementation with vitamin D3. No effects of vitamin D3 supplementation on serum leptin concentrations were found. Further research is still warranted to clarify the mechanisms behind the effect of vitamin D3 on these hormones. Future studies could consider adjusting for the use of antidepressant medications or exploring other factors that may influence the relationship between vitamin D and leptin/ghrelin. Cellular and molecular methods will be implicated in the future study to understand the interactions at different levels.

## Figures and Tables

**Table 1:** BDI scale.

Total score	Levels of depression
1–10	Considered normal.
11–17	Mild mood disturbance.
18–21	Borderline clinical depression.
22–31	Moderate depression.
32–40	Severe depression.
Over 40	Extreme depression.

**Table 2:** Demographic and anthropometric measurements of study participants (*n* = 72).

	Controls	MDD patients (baseline)	MDD patients (after vitamin D3 supplementation)
Gender (*n*)	20 males/21 females	8 males/32 females	
Age (years)	28.2 ± 1.02	42.5 ± 1.62	
Weight (kg)	73.3 ± 3.41	77.3 ± 3.00	76.1 ± 3.02
Height (kg)	166 ± 1.74	158 ± 1.46	158 ± 1.46
BMI (kg/m^2^)	26.3 ± 1.03	31.1 ± 1.30^∗∗∗^	30.7 ± 1.30^∗∗∗^

Data are presented as mean ± SEM. ∗∗∗*p* < 0.001 when compared with control. n: number; BMI: body mass index.

## Data Availability

Data are available upon reasonable request.
